# Effects of Ultrasonic Treatment on the Structure and Antioxidant Activity of Conjugates Formed by Porcine Blood Meal-Derived Peptides and Hemin

**DOI:** 10.3390/foods15122082

**Published:** 2026-06-08

**Authors:** Juanjuan Du, Xiaopeng Zhu, Jinxuan Cao, Jinpeng Wang, Yuemei Zhang, Wendi Teng, Ying Wang

**Affiliations:** Key Laboratory of Geriatric Nutrition and Health, School of Food and Health, Beijing Technology and Business University, Beijing 100048, China

**Keywords:** porcine blood meal, peptide, hemin, ultrasound, binding rate, spatial configuration, antioxidant activity

## Abstract

Porcine blood meal-derived hydrolysate peptides and hemin are natural antioxidants, and the formation of peptide–hemin conjugates can synergistically improve antioxidant performance. Ultrasonic (US) treatment facilitates the binding of different molecules. Therefore, in this study, the effects of ultrasonic power treatments on the antioxidant activity and binding behavior of peptide–hemin conjugates were investigated. The spatial structure of the peptide–hemin conjugates was characterized using endogenous fluorescence spectroscopy, Fourier transform infrared (FT-IR) spectroscopy, and circular dichroism (CD) spectroscopy, respectively. The results demonstrated that the peptide–hemin binding rate reached the highest value of 91.63% at 400 W US power, with structural changes in conjugates from α-helix to random coil structures. Additionally, US treatment increased the surface hydrophobicity and reduced the enthalpy change in conjugates. The antioxidant capacity was greatly improved and peaked at 400 W US, where DPPH and ABTS radical scavenging rates exceeded 55% and 65%, respectively. This study provided a scientific basis for the high-value utilization of US treatment on porcine blood meal resources.

## 1. Introduction

Porcine blood meal, a residual material produced throughout the meat processing industry, is rich in high-quality proteins and has great potential for high-value utilizations [1]. However, the current utilization of porcine blood meal remains relatively limited: it is mainly processed into feed, and a portion is even discarded as waste, leading to substantial resource depletion and environmental contamination [2,3]. Bioactive peptides derived from enzymatic hydrolysis of porcine blood proteins, as natural antioxidants derived from proteins, have attracted extensive attention due to their high safety, biocompatibility, and remarkable free radical scavenging ability [4]. Previous research screened and identified a series of antioxidant peptides successfully from the enzymatic hydrolysates of porcine blood meal. These peptides exhibited potent scavenging capacity against DPPH, ABTS, and hydroxyl radicals, and showed strong binding affinity with hemin—an iron-containing porphyrin compound that can enhance the antioxidant potential of bioactive substances through chelation or complexation [5]. Despite the promising antioxidant activity of the peptide–hemin conjugates, further optimization is required to fulfill the stringent requirements for high-potency antioxidants in practical applications.

Ultrasonic (US) treatment, a non-toxic, environmentally friendly non-thermal technology, maximizes nutrient retention [6] and facilitates dietary component interactions [7]. Due to its positive impact on the physical and functional properties of food [8,9], US treatment modifies food component properties to promote molecular binding and is increasingly applied in food processing, including enzymatic hydrolysis [10,11], drying of fruit and vegetable products [12], extraction of flavor peptides [13] and natural pigments [14]. As a mechanical vibration wave, US generates cavitation, shear forces, and microstreaming in liquid systems. These physical effects can disrupt non-covalent interactions within and between molecules, alter molecular conformation, and enhance intermolecular collisions, thereby promoting reactions and conjugation processes. For example, the US has been shown to improve the binding between proteins and sugars, and enhance the functional properties of their conjugates [15,16,17,18]. Previous studies have also demonstrated that US treatment significantly improved the antioxidant activity of bioactive peptides and their conjugates by modifying their molecular structure and increasing binding efficiency with functional ligands [19]. Building on these findings, we hypothesized that moderate US treatment can induce conformational unfolding of peptides, expose more binding sites, and promote coordination interactions between peptides and hemin. This, in turn, US treatment will enhance the formation of peptide–hemin conjugates and improve their antioxidant capacity.

This study adopted ultrasonic treatment to improve the antioxidant activity of antioxidant peptide–hemin conjugates. The spatial structure alterations of peptide–hemin conjugates under various US powers were characterized by ultraviolet-visible spectrum (UV-vis), endogenous fluorescence spectroscopy, Fourier transform infrared (FT-IR) spectroscopy, circular dichroism (CD) spectroscopy, and scanning electron microscopy. Additionally, differential scanning calorimetry (DSC) was used to explore the effects of different US powers on their thermal properties. This work aims to clarify the effect of US power pretreatments on peptide–hemin binding properties, thereby providing theoretical references for the efficient utilization of porcine blood meal resources and the application of US technology in the food industry.

## 2. Materials and Methods

### 2.1. Materials and Chemicals

Porcine blood meal used in this study was purchased from the Henan Daiyun Company (Zhengzhou, China). Potassium ferricyanide (99.5%), pyrogallol (98%), anhydrous ethanol (95%), DPPH (98%), ABTS (98%), 8-aniline-1-naphthalene sulfonic acid (ANS, 98%), KBr (99%), and hemin (98%) were all of analytical grade and supplied by Shanghai Yuanye Biotechnology Co., Ltd. (Shanghai, China). Throughout the experiments, deionized water was employed for all solution preparation and analytical procedures.

### 2.2. Preparation of Peptide–Hemin Conjugates

An aqueous suspension containing 10 wt% porcine blood meal solution was prepared, and the pH value was adjusted to 7.5 by 0.1 mol/L NaOH solution. Flavor protease (8000 U/g) was introduced to initiate enzymolysis, which proceeded for 5 h at a constant temperature of 55 °C. Upon completion of the reaction, the mixture was centrifuged at 8000× *g* for 10 min. The supernatant was then ultrafiltered with a 3 kDa tube, freeze-dried, and redissolved in deionized water prior to column chromatography. Chromatographic purification was performed on a Sephadex G-25 column at a flow velocity of 0.9 mL/min and a UV detection wavelength of 220 nm. Peptide fractions eluted within 70–80 min were harvested. The peptide fractions at a concentration of 1 mg/mL were mixed with 0.4 mg/mL hemin in a volume ratio of 3:1 (V_peptide_/V_hemin_) and allowed to react for 20 min.

### 2.3. Ultrasound Treatment

The peptide and hemin were mixed and then treated under US conditions (XM-700UHF; inner slot size: 500 mm × 300 mm × 150 mm; 22.5 L; US frequency, 80 KHz; power, 220 V; heating power, 500 W; Shanghai Jingxin Industrial Development Co., Ltd., Shanghai, China) for 20 min. The US powers were set as 0 W (CK), 100 W, 200 W, 300 W, 400 W, and 500 W, respectively. All samples were placed in an ice bath during ultrasonic processing to eliminate the interference of temperature elevation.

### 2.4. Binding Rate

The interaction between peptide fractions and hemin after US treatment was investigated using ultraviolet-visible (UV-Vis) spectroscopy (Tecan Infinite 200 PRO, Hombrechtikon, Swiss) and endogenous fluorescence spectroscopy (Hitachi F-4700, Tokyo, Japan). For UV-Vis analysis, the instrument was preheated for 15 min, and baseline calibration was conducted through deionized water, with a 200–500 nm scanning range. For fluorescence measurements, 10 μL of ANS solution (6 × 10^−3^ mol/L) was added to 2 mL of sample solution, followed by thorough mixing. The mixture was then kept in the dark for 15 min before analysis. The excitation wavelength was fixed at 350 nm, while the emission wavelength was fixed at 450–700 nm. The slit width was maintained at 5 nm during scanning. To determine the binding rate, peptide fractions and hemin were subjected to US treatment and allowed to interact for 20 min. Subsequently, 1 mol/L HCl solution was added to adjust pH, thereby precipitating the unbound hemin. The obtained supernatant was freeze-dried and weighed (M_0_). The binding rate was then calculated following Equation (1):

(1) $$Binding\;rate\;(\%)=\frac{0.4\times V_{hemin}-M_{0}}{0.4\times V_{hemin}}\times 100$$

where V_hemin_ presents the volume of hemin (mL); M_0_ presents the weight of supernatant freeze-dried powder (mg).

### 2.5. Surface Hydrophobic

The surface hydrophobicity of peptide–hemin conjugates subjected to different US power treatments was determined, referring to the previous method [20], with slight modifications. Fluorescent probe ANS was used to determine the surface hydrophobicity of samples using a Tecan Infinite 200 PRO spectrophotometer (Swiss). Prior to analysis, the samples were diluted to concentrations of 0.01, 0.02, 0.04, 0.06, and 0.08 mg/mL. 4 mL of each diluted solution was transferred into centrifuge tubes, followed by the addition of 20 μL ANS solution (8 mmol/L). After evenly mixing, the mixtures were maintained at room temperature and subjected to a light avoidance reaction for 10 min. Then, 200 μL of the sample solutions were loaded into a microplate to determine fluorescence intensity. The excitation and emission wavelengths were set at 390 nm and 470 nm, respectively, while the slit width was fixed at 5 nm. A linear regression curve was established by plotting fluorescence intensity against sample concentration. The initial slope obtained from the fitted curve was defined as the surface hydrophobicity index of the conjugates.

### 2.6. Determination of Structural Alteration

Structural changes in peptide–hemin conjugates treated under different US power conditions were characterized by Fourier transform infrared spectroscopy (FT-IR-4700, JASCO Inc., Tokyo, Japan) and circular dichroism spectroscopy (J-1500, JASCO Inc., Japan) with slight modifications [21]. After US treatment, the samples were freeze-dried prior to FTIR analysis. The dried powders were thoroughly blended with the standard potassium bromide at a mass ratio of 1:100 and then compressed into translucent pellets. Spectra were recorded over a wavenumber range of 400–4000 cm^−1^, with a resolution of 4 cm^−1^, while the standard potassium bromide served as the background reference. For circular dichroism measurements, 0.02 mg/mL sample solution was transferred into a quartz cuvette with a 0.1 cm path length. Spectra were collected in the range of 190–260 nm using a bandwidth of 1 nm. The deionized water was employed for baseline correction throughout the analysis.

### 2.7. Particle Size and Zeta Potential

The particle size of peptide–hemin conjugates was determined by the laser particle size analyzer (Malvern Mastersizer 2000, Malvern, UK). The zeta potential was measured by the ZetaSizer Nano ZS90 particle analyzer.

### 2.8. Determination of Antioxidant Capacity

#### 2.8.1. Dpph Clearance Rate

An aliquot of 2 mL sample solution (1 mg/mL) was transferred to a centrifuge tube, followed by the addition of 8 mL of 1 × 10^−4^ mol/L DPPH working solution. The resultant mixture was incubated in the dark for 30 min, then centrifuged at 4000× *g* for 6 min. The absorbance of the supernatant was measured at 517 nm (A_1_). For the blank sample (A_2_), 1 mL of sample solution was mixed with 4 mL 95% ethanol, and its absorbance was measured under the same conditions. Meanwhile, the absorbance of the control group (A_3_) was determined by combining 4 mL DPPH solution with 1 mL 95% ethanol. The DPPH clearance rate was calculated using Equation (2) [22]:

(2) $$DPPH\;clearance\;rate\%=\left(1-\frac{A_{1-}A_{2}}{A_{3}}\right)\times 100\%$$

where A_1_ represents the absorbance of the sample’s supernatant at 517 nm, A_2_ represents the absorbance of the blank sample’s supernatant at 517 nm, and A_3_ represents the absorbance of the control supernatant at 517 nm.

#### 2.8.2. Abts Free Radical Scavenging Rate

A 0.5 mL sample solution (1 mg/mL) was mixed with 2 mL ABTS solution in a centrifuge tube. The mixture was incubated in the dark for 15 min, and its absorbance (A) was measured at 734 nm. For the blank control, 0.5 mL of 95% ethanol was mixed with 2 mL ABTS solution, and the absorbance value (A_0_) was determined under the same conditions. The ABTS clearance rate was calculated using Equation (3) [22]:

(3) $$ABTS\;clearance\;rate\%=\frac{A-A_{0}}{A_{0}}\times 100\%$$

where A represents the absorbance of samples at 734 nm; A_0_ represents the absorbance of the blank control at 734 nm.

#### 2.8.3. Reducing Power

A 1 mL aliquot of the sample solution (1 mg/mL) was transferred into a test tube. Subsequently, 2.5 mL PBS solution (0.2 mol/L, pH 6.6) and 2.5 mL potassium ferricyanide solution (1%) were added in sequence, followed by thorough shaking to ensure proper mixing. The resulting mixture was incubated in a water bath maintained at 50 °C for 20 min. After completion of the reaction, the tubes were removed immediately. Subsequently, 2.5 mL TCA solution (10%) was added to terminate the reaction process. The mixtures were then subjected to centrifugation at 4000 rpm for 10 min. Following centrifugation, 1 mL of the supernatant was collected and combined with 1 mL ferric chloride solution (0.1%). Subsequently, 5 mL of distilled water was added, and the solution was mixed evenly before analysis. The absorbance was measured at 700 nm, and the higher the absorbance, the stronger the reducibility of the conjugates.

### 2.9. DSC

The thermal properties of the peptide–hemin were determined using a differential thermal scanner (DSC 3500 Sirius, NETZSCH, Selb, Germany), following the method of Malik [23]. The sample solution after US treatment was first freeze-dried, and then 2 mg of sample powder was weighed in a small aluminum crucible, sealed and pressed, and placed in the instrument. An empty crucible was placed on the left side as a control. The temperature was 25–200 °C, with a 10 K/s heating rate.

### 2.10. Statistical Analysis

Triplicate measurements were carried out for all experiments, and outcomes are presented as mean ± standard deviation. Shapiro–Wilk and Levene tests were used to verify normality and homogeneity of variance before statistics. One-way ANOVA coupled with the LSD post hoc test was performed using SPSS 19.0 to identify intergroup differences, and statistical significance was defined at *p* < 0.05. Graphical visualization of data was completed with Origin 2019b.

## 3. Results and Discussion

### 3.1. Analysis of Binding Rate

**Figure 1:**
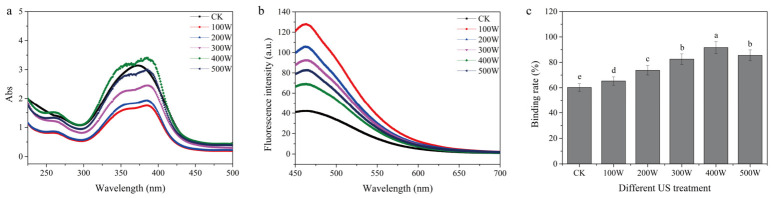
Ultraviolet-visible spectrum (**a**), fluorescence spectrum (**b**) and the binding rate (**c**) of peptide–hemin conjugates under different ultrasonic powers. Note: Different letters indicate significant differences between samples (*p* < 0.05).

UV-Vis absorption spectroscopy was employed to characterize the hemin-binding behavior of porcine blood hydrolysate peptides under varying US powers. As shown in Figure 1a, all spectra exhibited the characteristic Soret band of hemin, whose intensity and band profile directly reflected the formation and microenvironment stability of the peptide–hemin complexes [24]. Compared with the control (CK), low-power US (100–300 W) induced a transient decrease followed by a gradual recovery in Soret absorbance. Quantitatively, the absorbance at 300 W decreased to 0.54 times the control value, indicating insufficient peptide unfolding and limited hemin accessibility at low ultrasonic energy. At 400 W, the absorbance reached its maximum (1.16 times the control value), which may be ascribed to the exposure of buried hydrophobic groups and chromophores following structure unfolding, leading to the formation of more stable complexes [25]. By contrast, absorbance declined again at 500 W, suggesting that excessive power may cause partial peptide degradation or complex disassembly, compromising the binding efficiency. Collectively, these results demonstrate that 400 W is the optimal US power to promote the formation of stable peptide–hemin complexes.

The fluorescence spectra in Figure 1b showed that US treatment significantly enhanced the fluorescence intensity of the conjugates. With the enhancement of US power, the fluorescence intensity of the conjugates exhibited a pattern of initial decrease followed by an increase. At 400 W US, the fluorescence intensity of the conjugates reached the lowest level. US treatment modified molecular conformation, rearranged chromophore groups, exposed them to the solvent environment, altered local environmental polarity, and induced intermolecular aggregation, which collectively accounted for the reduced fluorescence intensity of the conjugates [26]. Consistent with our findings, Zhao et al. also found that partial protein particle aggregation re-embedded tryptophan inside protein aggregates and decreased fluorescence intensity [27].

The binding rates of peptide and hemin at various US powers were subsequently determined, with the result presented in Figure 1c. Compared with the control group, US treatment significantly improved the peptide–hemin binding rate (*p* < 0.05). When the US power was below 400 W, the binding rate of peptide–hemin increased significantly with the increasing US power (*p* < 0.05), and reached the highest (91.63%) at 400 W. Further increasing the power to 500 W reduced the binding affinity of peptide–hemin, possibly due to weakened interactions and excessive aggregation induced by excessive ultrasonic intensity [28]. This was consistent with the research of Zhao et al., showing that moderate US treatment could significantly enhance the binding affinity between molecules [27]. Similarly, Cao et al. also demonstrated that US treatment altered the distribution of proteins and small molecules, promoting molecular interaction and stability of conjugates [29]. Such US-promoted molecular binding concurrently enhanced the antioxidant properties of the conjugates.

### 3.2. Surface Hydrophobicity

**Figure 2:**
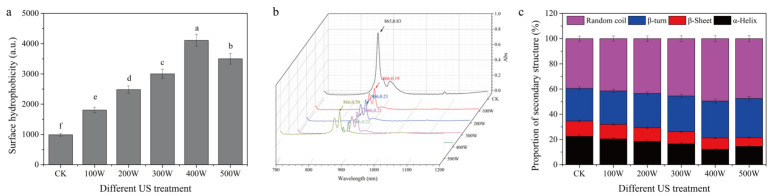
The surface hydrophobicity (**a**), Fourier transform infrared spectrum (**b**) and secondary structure content (**c**) of peptide–hemin conjugates under different ultrasonic powers. Note: Different letters indicate significant differences between samples (*p* < 0.05).

Surface hydrophobicity is one of the structural characteristics utilized to evaluate the effect of conformational alterations on functional properties [21]. The surface hydrophobicity of the peptide–hemin conjugates under different US powers was shown in Figure 2a. Compared with the non-ultrasonic group, US treatment significantly increased the surface hydrophobicity (*p* < 0.05) with increasing power, peaking at 400 W before decreasing at 500 W. This suggested that US treatment induced partial unfolding of peptide spatial structure, thereby exposing more hydrophobic groups and regions to the polar environment [30]. The result coincided with Hu et al., who attributed elevated protein surface hydrophobicity to US cavitation forces and microfluidic effects [15]. Significant differences in surface hydrophobicity among US groups indicated varying US powers caused different levels of buried hydrophobic group exposure [21]. As reported by Chen et al., strong ultrasonic cavitation destroyed initial peptide structure and non-covalent interactions (e.g., hydrophobic interactions, electrostatic forces, and hydrogen bonds), exposing peptide hydrophobic groups and enhancing hydrophobicity between components [31]. Once the US power > 400 W, the surface hydrophobicity of the conjugates decreased, which might be because high-power US pretreatment destroyed the structure and caused conjugates to aggregate, resulting in the re-inclusion of hydrophobic groups. Notably, the trends in surface hydrophobicity matched the binding rate in our research, which is similar to the findings of previous studies. That is, when the binding rate increased, the surface hydrophobicity of the conjugates also increased [32].

### 3.3. Structural Alteration Analysis

The FT-IR spectra of peptide–hemin conjugates in the 700–1200 cm^−1^ region under varying US power levels are presented in Figure 2b. A prominent characteristic absorption band was observed at approximately 865–866 cm^−1^, which is mainly attributed to the out-of-plane deformation vibration of carboxyl groups in peptide chains and is closely related to the coordination interaction between peptides and hemin [5]. With increasing ultrasonic power from 100 to 400 W, the absorbance at this band decreased gradually, indicating that low-intensity US disrupted the intrinsic molecular interactions and altered the microenvironment of carboxyl groups. As the power increased to 300–400 W, the spectral changes became more pronounced, suggesting that moderate ultrasonic cavitation and mechanical forces induced peptide unfolding and conformational rearrangement, thereby exposing more carboxyl groups and promoting the formation of new coordination bonds with hemin [33]. When the ultrasonic power further increased to 500 W, excessive US might cause peptide degradation or irreversible aggregation, which disrupted the interaction balance between peptides and hemin. Overall, the absorbance intensity clearly demonstrated that US treatment modulated the non-covalent interactions and coordination states of peptide–hemin conjugates.

US parameters affected the secondary structure of proteins or peptides [34]. Figure 2c depicts the effect of US treatment on the alterations in the secondary structure of the conjugates, with obvious structural discrepancies observed across different power levels. At 400 W US, the α-helix content was the lowest, whereas the random coil content reached its highest point. The transformation of α-helix structure to random coil demonstrated that US altered the secondary structure of conjugates, which was attributed to US cavitation-induced molecular unfolding, local conformational rearrangement to random coil [35]. Consistent with our results, Cheng et al. also found that US could lead to a decrease in α-helix and an increase in random coil, thereby exposing more hydrophobic residues [36]. At 400W US power, the transformation of secondary structure promoted the binding between peptides and hemin, which aligns with the conclusions drawn by Li et al. that US improved the degree of conjugation with reduced α-helix content but increased random coil structures, along with enhanced surface hydrophobicity [37]. Furthermore, Chen et al. reported that a high degree of random coil was accompanied by high surface hydrophobicity and high antioxidant properties of conjugates [38], laying the foundation for explaining the changes in the antioxidant properties of the peptide–hemin conjugates.

### 3.4. Particle Size and Zeta Potential Analysis

**Figure 3:**
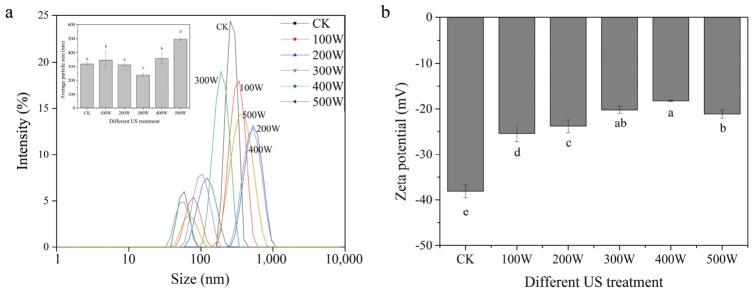
The particle size (**a**) and zeta potential (**b**) of peptide-hemin conjugates of each ultrasonic treatment group. Note: Different letters indicate significant differences between samples (*p* < 0.05).

The particle size of the peptide–hemin conjugates under varying US power levels is illustrated in Figure 3a. After US treatment, the average particle size of the conjugates showed an up–down–up tendency after US treatment. Low-intensity US did not damage protein molecules, but induced their regular arrangement to form unstable aggregates and increase particle size. As the US continues, the particle size decreased, and the unstable aggregates were violently decomposed into smaller aggregates by the physical forces of US (such as shear force, micro-stream flow, and shock waves). In higher-intensity ultrasound treatment, the small particles underwent re-aggregation, resulting in an increase in particle size [34]. These findings agreed with Zhao et al. (2022), who claimed that moderate US reduced particle size, while an increase in particle size under the ultrahigh US power was due to an increase in the hydrophobic interactions and aggregation between molecules [27]. Existing literature has also confirmed that US can further influence the antioxidant activity of conjugates by altering the interaction mode and intensity between molecules, as well as the size of the molecules [39].

The zeta potential is commonly used to characterize the surface charge properties of particles and can reflect the electrostatic interaction occurring between them [40]. As shown in Figure 3b, peptide–hemin conjugates exhibited negative zeta potential values across the entire US power range tested (0–500 W), indicating that negative charges on the peptide–hemin conjugates’ surface were consistently higher than positive charges under all treatment conditions [27]. Additionally, the absolute value of zeta potential decreased gradually with increasing US power, indicating the decreased stability of the conjugates and the tendency to aggregate [41]. The changes in zeta potential might be attributed to US-induced structural alterations of the conjugates, leading to the exposure of more positively charged amino acid residues [42]. This finding confirmed that the US treatment altered the conformation and promoted the surface residue distribution, which was highly in agreement with the results obtained from the aforementioned multispectral technique. While the reduced surface charge weakens particle stability, the structural changes induced by US bring positive outcomes for the antioxidant function of the conjugates. A study by Shao et al. has reported that US-induced conjugates exhibited a decrease in absolute zeta potential and an increase in antioxidant properties, which is beneficial for enhancing digestibility [43].

### 3.5. Analysis of Antioxidant Activity

**Figure 4:**
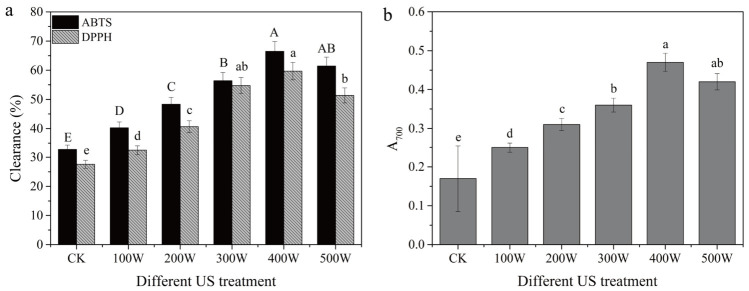
DPPH clearance rate and ABTS clearance rate (**a**), reducing capacity (**b**) of peptide–hemin conjugates under different ultrasonic powers. Note: Different letters indicate significant differences between samples (*p* < 0.05).

2,2-Diphenyl-1-picrylhydrazyl (DPPH) and ABTS radical scavenging assays, as well as reducing power measurement, are used to evaluate the antioxidation capacity of peptide–hemin conjugates. As shown in Figure 4. compared with the control group, the DPPH scavenging rate, ABTS clearance, and reducing power of the US-treated groups significantly increased with the rise in US power (*p* < 0.05), reaching the maximum values at 400 W. Specifically, the ABTS clearance rate exceeded 65%, and the DPPH clearance rate exceeded 55%, which might be attributed to the structural modification and unfolding of peptides, as well as the exposure of antioxidant amino acids within the peptides [44]. Nevertheless, further increasing the US power to 500 W caused a notable decline in the DPPH, ABTS scavenging activity and reducing power. This was consistent with the variations in the binding rate of peptide–hemin under different US power treatments. In agreement with our findings, Xu et al. also discovered that high-intensity US treatment reduced the antioxidant properties of conjugates [44]. The mechanical force generated by moderate US may loosen the structure of peptides, which further facilitates the release of antioxidant peptides [45]. In general, US treatment altered the binding mode of peptide–hemin conjugates and further regulated their antioxidant capacity. The variations in antioxidant activity observed in this study were closely related to the structural changes in the conjugates induced by US.

### 3.6. Analysis of Thermal Characteristic

**Figure 5:**
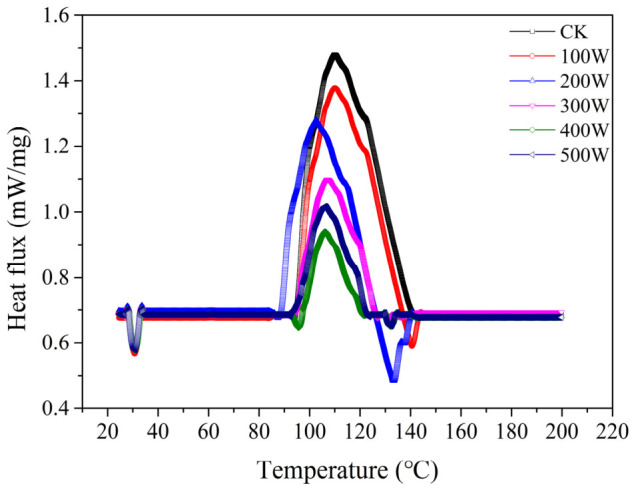
DSC of peptide–hemin conjugates under different ultrasonic treatment groups.

Differential scanning calorimetry (DSC) is used to characterize the thermal properties and thermal reactions of food materials. Through the thermal transition temperature (Tp) and enthalpy change corresponding to the thermal transition, moisture and protein in food materials can be evaluated [46]. Figure 5 shows the DSC variations of peptide–hemin conjugates under different US power treatments. All treatment groups exhibited a single-peak exothermic curve, indicating that conjugates underwent a uniform exothermic reaction within the test temperature range. As US power increased, both Tp and enthalpy change values decreased, reaching their minimum at 400 W, followed by a slight elevation at 500 W. Combined with the secondary structure results, such thermal variations might be ascribed to US-induced conformation modification of the conjugates, which affected their thermal properties [47]. The decreased Tp indicated partial unfolding of the conjugate structure and consequent higher susceptibility to heat-induced denaturation, which was likely attributed to the disruption of non-covalent interactions and exposure of internal amino acid residues induced by acoustic cavitation [48]. Consistent with the present findings, Cimen et al. also found that US reduced the thermal denaturation temperature of meat proteins [47]. Notably, the low thermal denaturation temperature is beneficial for digestion [48], providing a favorable basis for the application of peptide–hemin conjugates after US treatment in food products. In conclusion, increasing US power modulated the peptide–hemin binding, as well as the exposure of specific amino acid residues, thereby remodeling the peptide conformation and ultimately altering the thermal properties of the conjugates.

## 4. Conclusions

This study systematically explored the impacts of US treatment on the conformational structure and antioxidant capacity of peptide–hemin conjugates derived from porcine blood meal. The binding efficiency of the conjugates exhibited significant power-dependent variations under US irradiation, with the maximum binding rate of 91.63% achieved at an optimal US power of 400 W. Relative to the untreated control (CK) group, 400 W US treatment triggered prominent conformational rearrangement of the conjugates, characterized by the conversion of α-helix structures into random coils. Such structural modifications were concurrently associated with elevated surface hydrophobicity, improved antioxidant performance, reduced absolute zeta potential values, as well as declined melting temperature Tp and enthalpy changes. Collectively, this work establishes a mechanistic foundation for the high-value utilization of porcine blood meal by-products and further expands the application potential of ultrasonic technology in the food processing industry.

Nevertheless, several important limitations of the current research should be clearly addressed. First, structural analysis was mainly based on conventional FT-IR, CD, particle size and zeta-potential measurements; advanced quantitative characterizations including LC-MS/MS, MALDI-TOF, XPS, Raman, SEC-HPLC and electron microscopic observation (SEM/TEM) were not performed, so the interpretation of structure–antioxidant correlation remains correlational rather than mechanistic, lacking direct quantitative evidence for hydrogen bond alteration, specific amino acid residue exposure, peptide degradation and irreversible aggregation induced by excessive high-power ultrasound. Second, antioxidant capacity was only determined via classic in vitro chemical assays (DPPH, ABTS and reducing power), while biologically relevant antioxidant models such as the ORAC test, protein oxidation detection and long-term storage oxidative stability evaluation were not included. Third, all trials were implemented in simple aqueous solution systems; we failed to investigate the physicochemical variation and functional behavior of conjugates within complex real food matrices, as well as the influences of particle size and zeta potential changes on their actual bioavailability and in-food functional stability. Based on the above limitations, future research will introduce multiple advanced instrumental analyses to deeply clarify molecular interaction mechanisms. Additional biological antioxidant assessments and storage stability tests will be arranged, and follow-up trials will focus on conjugate application performance in various food systems. Moreover, pilot-scale experiments will be carried out to evaluate economic cost and processing feasibility, so as to accelerate the industrial translation of this ultrasonic modification technique.

## Data Availability

The data presented in this study are available on request from the corresponding author.
